# Discrimination of the moving direction is improved depending on the pattern of the mechanical tactile stimulation intervention

**DOI:** 10.1186/s12868-024-00855-2

**Published:** 2024-03-05

**Authors:** Yuki Maruyama, Sho Kojima, Hideaki Onishi

**Affiliations:** 1 Graduate School, Niigata University of Health and Welfare, 1398 Shimami-cho, Kita-Ku, 950-3198 Niigata City, Niigata Japan; 2 Institute for Human Movement and Medical Sciences, Niigata University of Health and Welfare, 1398 Shimami-cho, Kita-Ku, 950-3198 Niigata City, Niigata Japan; 3 Department of Physical Therapy, Niigata University of Health and Welfare, 950-3198 Niigata City, Niigata Japan

**Keywords:** Mechanical tactile stimulation, Moving direction, Reaction time, Correct rate, Rate correct score

## Abstract

**Background:**

The mechanical tactile stimulation, such as plastic pins and airflow-driven membrane, induces cortical activity. The cortical activity depends on the mechanical tactile stimulation pattern. Therefore, the stimulation pattern of mechanical tactile stimuli intervention may influence its effect on the somatosensory function. However, the effect of the mechanical tactile stimulation input pattern on the somatosensory function has not yet been investigated at the behavioral level. The present study aimed to clarify the effects of mechanical tactile stimuli intervention with different stimulation patterns on the ability to discriminate moving directions.

**Results:**

Twenty healthy adults participated in the experiment. Three conditions were used for mechanical tactile stimuli intervention: (1) the whole stimulus surface was stimulated, (2) the stimulus moved within the stimulus surface, and (3) a no-stimulus condition. The effects of mechanical tactile stimuli intervention on tactile discrimination were evaluated using a simple reaction task and a choice reaction task to discriminate the movement direction. Reaction time, correct rate, and rate correct score were calculated to measure task performance. We examined the effects of mechanical tactile stimuli intervention on the ability to discriminate the moving direction for a certain period under three intervention conditions. The results showed that the mean reaction time during the simple reaction task did not differ significantly before and after the intervention under all intervention conditions. Similarly, we compared the data obtained before and after the intervention during the choice reaction task. Our results revealed that the mean reaction time and correct rate did not differ significantly under vertical and horizontal conditions. However, the rate correct score showed a significant improvement after the horizontal moving tactile stimulation intervention under both vertical and horizontal conditions.

**Conclusions:**

Our results showed that the effect of mechanical tactile stimuli intervention on mechanical tactile stimulation moving direction discrimination function depended on the input pattern of mechanical tactile stimuli intervention. Our results suggest the potential therapeutic benefits of sustained tactile stimulation intervention. This study revealed that it is possible to change behavioral levels via mechanical tactile stimuli intervention as well as the potential of mechanical tactile stimuli intervention in the field of rehabilitation.

## Background

Somatosensory stimulus input provides various information. Previous studies have focused on the ability to discriminate various stimulus parameters, including frequency [1–3], time [4, 5], intensity [6], space [5, 7], pattern [8, 9], and motion [10–13]. When discriminating stimuli motions in two directions, a previous study reported that the stimuli presented to the fingertip were discriminated beyond the chance level between the stimulus that moved parallel to the long axis of the finger (vertical) and that across the finger (horizontal) [10]. Previous studies using fMRI showed that there is significant activity in the primary somatosensory cortex (S1), secondary somatosensory cortex (S2), and inferior parietal cortex (IPC) during the task of discriminating the direction of movement of stimuli motion [11]. Thus, the activity in these cortical regions is possibly important in discriminating the moving direction of somatosensory stimulation.

Mechanical tactile stimulation (MS) is one of the somatosensory stimuli, and cortical excitability and somatosensory function are changed after high-frequency MS interventions [14–19]. Repetitive stimuli have been reported to induce long-term potentiation (LTP) when the input is at a high frequency of ≥ 5 Hz [20–22]. In the MS intervention, 20 min of high-frequency MS intervention (20 Hz) was suggested to induce LTP-like changes and to improve two-point discrimination perception [17, 18]. Furthermore, the changes in cortical excitability with the MS intervention depend on the stimulus pattern [19, 23, 24]. In a 20-minute MS intervention (20 Hz; 1 s on, 5 s off) using plastic pins, the changes in somatosensory-evoked magnetic fields depended on the pattern of the MS intervention with moving the row of pins within the stimulation plane or repeated stimulation of the finger pad [19]. Therefore, the stimulus pattern of the MS intervention may influence the effect of the intervention on the somatosensory function; however, the effect of the input pattern of the MS intervention on the somatosensory function has not been investigated at the behavioral level.

The differences in MS patterns were reported to evoke activity in different cortical regions [25, 26]. For example, MS moving in one direction on the finger pad showed significant activity in S1 and S2 as compared to MS without movement, and MS moving regularly on the finger pad showed a significant activity in the IPC as compared to moving randomly [25]. Consequently, MS moving regularly in one direction induced activity in the cortical regions involved in discriminating the moving direction of MS. Thus, the high-frequency MS input moving regularly in one direction may activate cortical regions involved in discriminating the direction of MS movement and improve somatosensory discrimination function. However, the effects of MS interventions on somatosensory functions that are dependent on MS input patterns were not examined.

The present study aimed to investigate the effects of the MS interventions with different stimulus patterns on somatosensory discrimination functions at the behavioral level using the MS moving direction discrimination task. MS moving regularly in one direction induces activity in cortical regions involved in discriminating the moving direction of MS. Accordingly, we hypothesized that the intervention with high-frequency MS input moving in one direction improves MS’ ability to discriminate the motion direction. The present study expands the knowledge regarding the effect of MS interventions depending on the input pattern.

## Results

### Choice reaction task

**Fig. 1:**
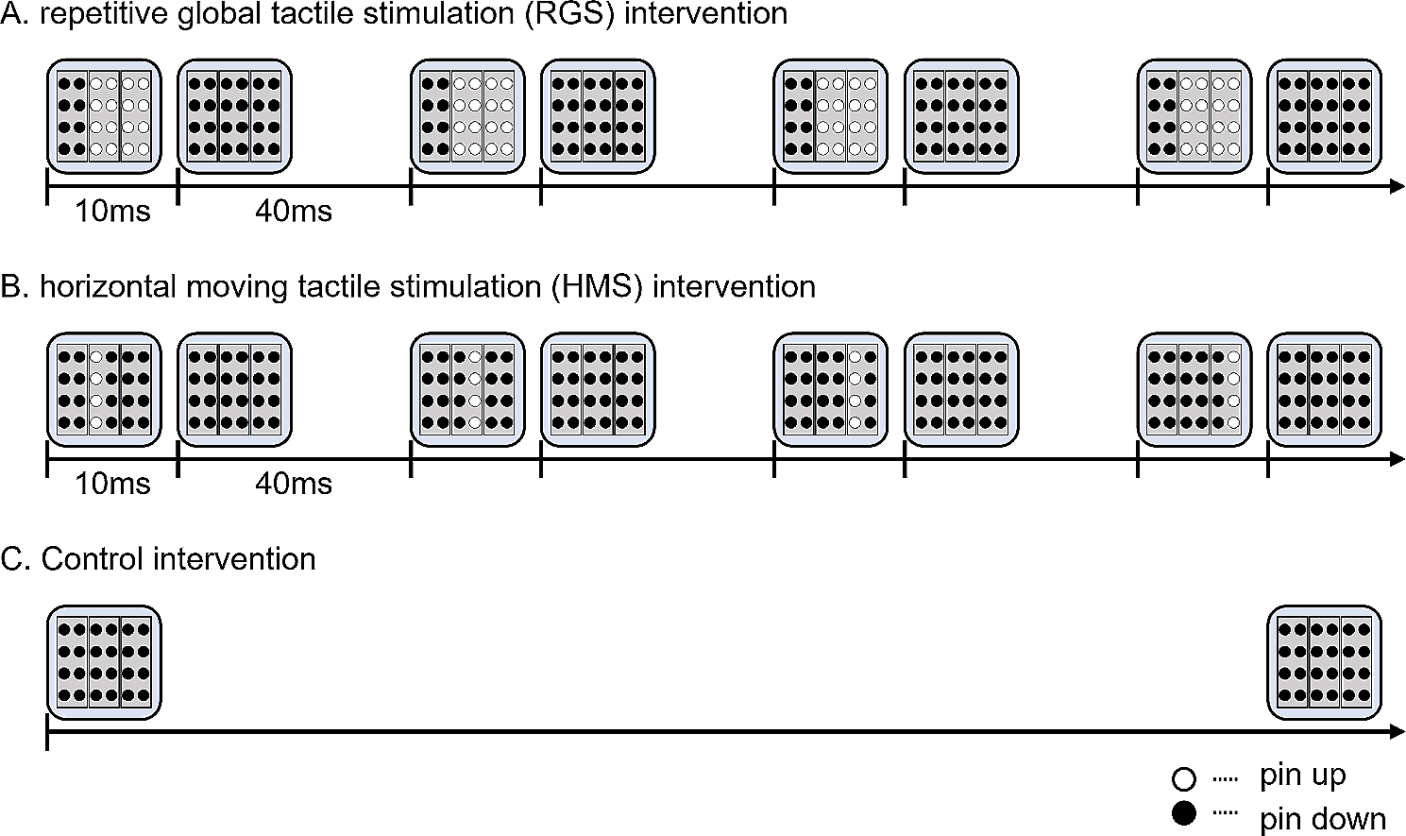
Stimulus paradigm each mechanical tactile stimulation intervention conditions. The three intervention conditions were as follows: (**A**) repetitive global tactile stimulation (RGS) intervention, in which all 16 pins appeared and disappeared at the same time; (**B**) horizontal moving tactile stimulation (HMS) intervention, in which a row of four vertically aligned pins moved and from left to right before it disappeared; and (**C**) Control intervention, in which the right index finger is placed on the device without stimulation

Three MS interventions were employed in the current study. The repetitive global tactile stimulation (RGS) intervention stimulated the index finger with all 16 pins at the same time (Fig. 1A), the horizontal moving tactile stimulation (HMS) intervention stimulated the index finger with a row of four vertically aligned pins moved from left to right (Fig. 1B), and the Control intervention, the right index finger was placed on the device without any stimulation (Fig. 1C).

**Fig. 2:**
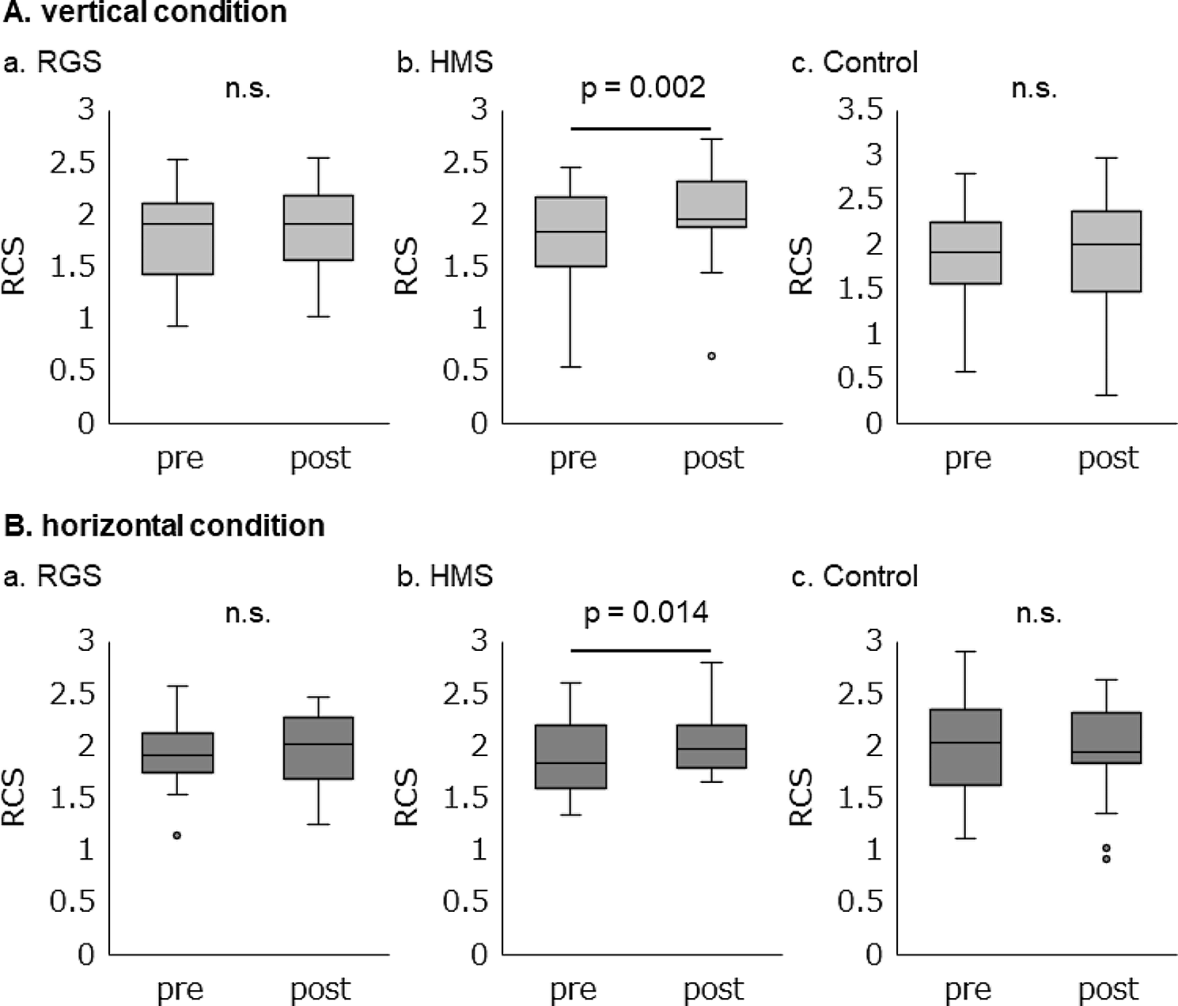
Rate correct score of pre and post mechanical tactile stimulation intervention. The rate correct score (RCS) for the choice reaction tasks. Comparison of the RCS before the intervention with that after the intervention in the (**A**) vertical and (**B**) horizontal conditions. (**a**) RGS intervention. (**b**) HMS intervention. (**c**) Control intervention. n.s: not significant

We used the Wilcoxon signed-rank test to compare the RCS before and after the intervention. In the vertical condition of the HMS intervention, the RCS shifted 1.8 ± 0.5 to 2.0 ± 0.4. The Wilcoxon signed-rank test revealed that RCS was significantly higher after the intervention than before the intervention (*p* = 0.002, *r* = 0.684) (Fig. 2Ab). However, the Wilcoxon signed-rank test revealed that RGS and Control interventions showed no significant difference before and after the intervention (RGS intervention: *p* = 0.455, *r* = 0.167; Control intervention: *p* = 0.296, *r* = 0.234) (Fig. 2Aa, c). The RCS shift in the RGS intervention was 1.8 ± 0.4 to 1.8 ± 0.4, and that in the Control intervention was 1.9 ± 0.5 to 1.9 ± 0.6. In the horizontal condition of the HMS intervention, the RCS shifted from 1.9 ± 0.4 to 2.0 ± 0.3. The Wilcoxon signed-rank test revealed that the RCS was significantly higher after the intervention than before the intervention (*p* = 0.014, *r* = 0.551) (Fig. 2Bb). In contrast, the Wilcoxon signed-rank test revealed that RGS and Control interventions showed no significant differences before and after the intervention (RGS intervention: *p* = 0.167, *r* = 0.309; Control intervention: *p* = 0.263, *r* = 0.250) (Fig. 2Ba, c). The RCS shift in the RGS intervention was 1.9 ± 0.4 to 2.0 ± 0.3, and that in the Control intervention was 2.0 ± 0.5 to 2.0 ± 0.5.

We used a generalized linear mixed model (GLMM) to analyze the mean RTs and correct rate. The mean reaction times (RTs) and correct rate are shown in Table 1. The mean RTs in the vertical condition showed no significant main effect of the intervention condition (RGS, HMS, and Control intervention) (F (2, 95) = 0.063, *p* = 0.939) and interaction effect (F (2, 95) = 0.740, *p* = 0.480). Contrarily, there was a significant main effect of time (pre, post) (F (1, 95) = 11.510, *p* = 0.001). In the horizontal condition, no significant main effect of the intervention condition (F (2, 95) = 0.357, *p* = 0.701) and interaction effect (F (2, 95) = 0.164, *p* = 0.849) were observed. Contrarily, a significant main effect of time was seen (F (1, 95) = 6.200, *p* = 0.015).

**Table 1:** Changes in the mean RT and correct rate before and after the intervention

	**Choice reaction task (vertical condition)**						
	RGS		HMS		Control		
	**pre**	**post**	**pre**	**post**	**pre**	**post**	
Mean RT	504.3 ± 67.7	487.7 ± 73.9	521.1 ± 104.6	478.9 ± 74.5	520.5 ± 113.0	478.3 ± 89.2	
(ms)							
Correct rate	87.2 ± 15.0	86.6 ± 13.5	89.6 ± 14.6	93.2 ± 12.6	91.1 ± 13.0	84.8 ± 21.2	
(%)							
	**Choice reaction task (horizontal condition)**						
	RGS		HMS		Control		
	**pre**	**post**	**pre**	**post**	**pre**	**post**	
Mean RT	488.1 ± 56.1	471.6 ± 54.3	504.6 ± 86.5	475.0 ± 66.9	497.4 ± 100.1	471.8 ± 75.5	
(ms)							
Correct rate	91.3 ± 10.7	92.9 ± 9.7	94.7 ± 4.3	95.1 ± 4.6	94.0 ± 7.8	90.7 ± 14.1	
(%)							
	**Simple reaction task**						
	RGS		HMS		Control		
	**pre**	**post**	**pre**	**post**	**pre**	**post**	
Mean RT	203.4 ± 39.2	202.3 ± 27.5	202.5 ± 28.2	208.4 ± 29.6	197.6 ± 37.7	207.7 ± 51.5	
(ms)							
Correct rate	−	−	−	−	−	−	
(%)							

Mean ± standard deviation

The correct rate in the vertical condition showed no significant main effect of intervention condition (F (2, 95) = 1.533, *p* = 0.221), time (F (1, 95) = 0.251, *p* = 0.617), and interaction effect (F (2, 95) = 1.708, *p* = 0.187). Further, in the horizontal condition, no significant main effect of the intervention condition (F (2, 95) = 1.529, *p* = 0.222), time (F (1, 95) = 0.090, *p* = 0.765), and interaction effect (F (2, 95) = 1.038, *p* = 0.358) was noted.

### Simple reaction task

The mean RTs in the simple reaction task (SRT) before and after the intervention are shown in Table 1. The mean RTs was compared using GLMM. There was no significant main effect of the intervention condition (RGS, HMS, and Control intervention) (F (2, 95) = 0.197, *p* = 0.821) and time (pre, post) (F (1, 95) = 1.512, *p* = 0.222) and interaction effect (F (2, 95) = 0.645, *p* = 0.527).

## Discussion

In the present study, we investigated the effects of the MS intervention for a certain time with different stimulation patterns on the ability of discrimination of the moving direction. The results showed that the mean RTs in the SRT did not change significantly before and after the intervention in all intervention conditions. Similarly, we compared the data obtained before the intervention with those obtained after the intervention in the CRT. Our results showed that the mean RTs and correct rate were not significantly changed in the vertical and horizontal conditions. However, the RCS had a significant improvement after the intervention only in the HMS intervention in both vertical and horizontal conditions. Therefore, the moving tactile stimulation intervention for a certain time improves the ability to discriminate moving directions.

There were no significant changes in the mean RTs of the SRT before and after the intervention in all intervention conditions. In a previous study, compared with before practice, the RT was significantly shorter after practicing SRT 60 times for 3 days, with 6 sets per day [27]. In the present study, the SRT was performed only at the time of evaluation, and each intervention was conducted at least every 5 days; thus, the RT was not reduced unlike in previous studies.

In CRT, both the mean RTs and correct rate did not change significantly after the intervention, as compared with that before the intervention. This could be due to a “speed-accuracy tradeoff.” A “speed-accuracy tradeoff” is defined when the “speed” of the reaction is emphasized, wherein the reaction speed increases, but the error rate is high. Contrarily, when “accuracy” is emphasized, the error rate is lowered, but the reaction speed decreases. This “speed-accuracy tradeoff” may have resulted in a different balance between “speed” and “accuracy” among individuals. Therefore, no significant change in the mean RTs and correct rate could have been observed.

The Wilcoxon signed-rank test results showed that the RCS did not change significantly after the intervention as compared with that obtained before the intervention in the RGS and Control interventions. Contrarily, only the HMS intervention significantly increased the RCS after the intervention as compared with that before the intervention. This result may suggest that the HMS intervention might improve direction discrimination of moving MS. The reason why RCS was only improved in the HMS intervention is possibly due to the fact that the different stimulation patterns evoke cortical activities in different regions. Previous studies on the effects of MS have shown that cortical activity changed in an input pattern-dependent manner [25, 26]. In a previous study that used functional magnetic resonance imaging, cortical activity was compared immediately after the input of moving MS and stationary MS. The results showed that S1 and S2 were significantly more active in the moving MS as compared to those in the stationary MS. Additionally, the cortical activity was compared immediately after the input of a regularly moving MS and a randomly moving MS. The results showed that S1, S2, IPC, and hMT+/V5 were significantly more active in the regularly moving MS than in the randomly moving MS [25]. The HMS intervention used in this study involved repeated presentations of regularly moving stimulation, and thus, it is expected to elicit an increased activity in the abovementioned cortical regions.

Somatosensory information from the periphery is transmitted from the sensory receptors in the skin to the S1 of the contralateral hemisphere via the spinal cord and thalamus. In previous studies, magnetoencephalography showed S1 activation after applying somatosensory stimulation to the skin [28–30]. Somatosensory information is thought to be processed in stages, first in S1, followed by downstream stages in S2 and posterior parietal cortex, to which S1 transmits information directly [31, 32]. In addition to these core somatosensory cortical regions, a variety of other cortical regions are active during the discrimination task under MS [8, 9, 33]. Regarding the tactile direction discrimination used in our previous study, the right index and middle fingers were placed on separate rails, and the task was to discriminate between congruent or incongruent directions, in which pins moved on the rails; the results revealed that S1, S2, and IPC were active during this task [11]. In another study, a moving tactile stimulation was provided to both index fingers simultaneously to the right or left. The participants were asked to discriminate the right or left movement and reacted by pressing a button; S1, S2, IPC, and hMT+/V5 were active during this task [12]. In the present study, in the CRT, the participants were presented with a tactile stimulation moving vertically or horizontally on the right index finger, and they reacted to the moving direction by pressing a button with their left hand. The activity of S1, S2, IPC, and hMT+/V5 involved with the CRT may be similar to that previously reported [11, 12]. Therefore, the HMS intervention was thought to enhance the activity of cortical regions that discriminate MS moving in one direction, thereby improving the RCS in both horizontal and vertical conditions.

One limitation of the present study is that the cortical activity before and after the intervention was not recorded; thus, it is uncertain whether the MS intervention changes the activity of higher cortical regions involved in sensory information processing. Future studies should record the cortical activity before, during, and after the intervention to clarify its effects on cortical excitability.

## Conclusions

Our data revealed that the MS intervention with a unidirectional regularly moving tactile stimulus improved direction discrimination sensitivity of somatosensory function.

## Methods

### Participants

Twenty right-handed healthy volunteers (mean ± standard deviation, 20.9 ± 0.3 years; 10 men and 10 women) participated in this experiment. No participants were taking medications that were known to affect the central nervous system prior to the experiment. The experiment was conducted after obtaining the patients’ informed consent. The present study was approved by the Ethics Committee of Niigata University of Health and Welfare (18157–190703) and was conducted in accordance with the guidelines stipulated in the Declaration of Helsinki.

### Experimental design

**Fig. 3:**
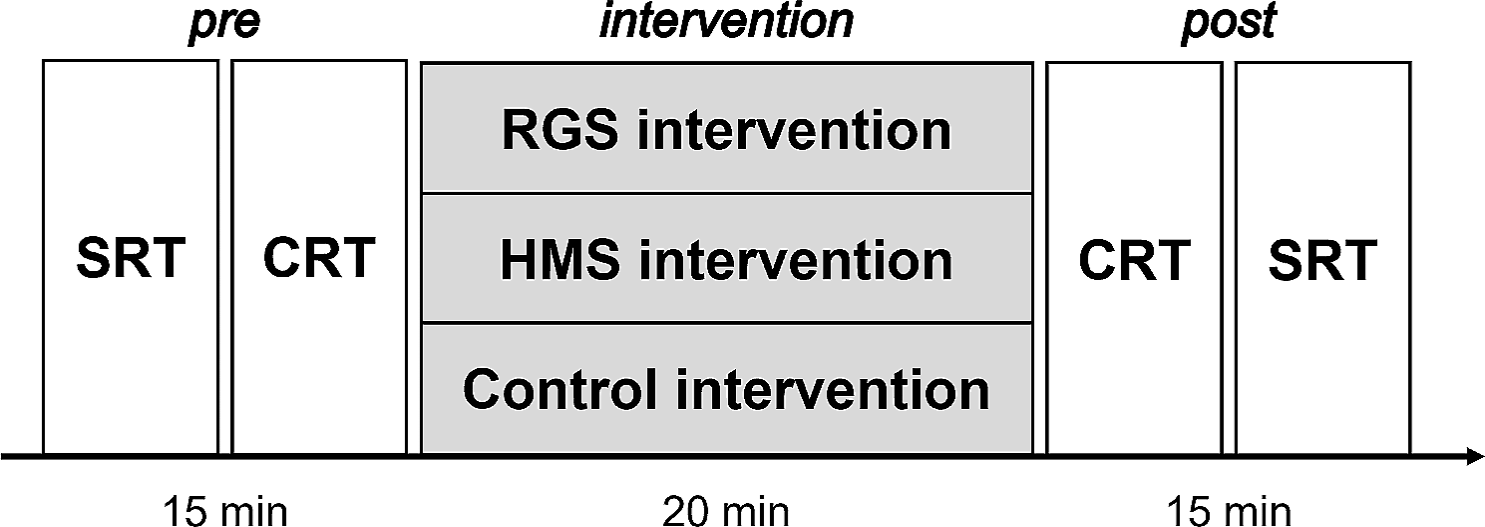
Experimental protocol. The study’s experimental protocol. The simple reaction task (SRT) and choice reaction task (CRT) were assessed pre- and post-intervention in the order shown in the figure. One of the three intervention conditions was presented for 20 min. The interval between each condition was at least 5 days. RGS: repetitive global tactile stimulation; HMS: horizontal moving tactile stimulation

The participants were identified as right-handed using the Edinburgh Handedness Inventory [34]. The experiment was started after explaining the task verbally. The experimental protocol is shown in Fig. 3. The participants performed a SRT and CRT before the intervention. Subsequently, one of the three intervention conditions was presented for 20 min. They performed SRT and CRT again after the intervention.

### Simple reaction and choice reaction tasks

**Fig. 4:**
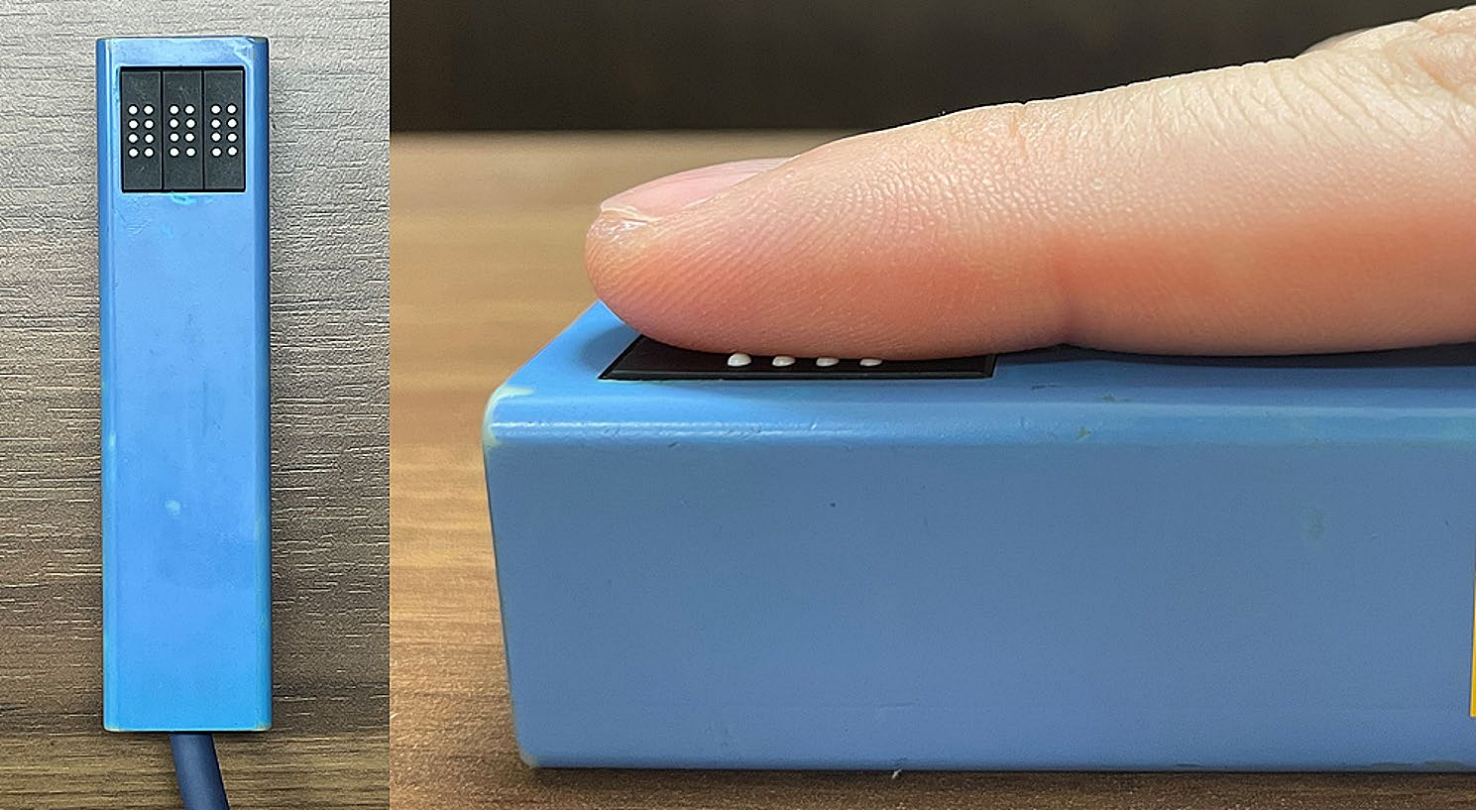
Presentation of mechanical tactile stimulation. This study used a piezoelectric tactile stimulator. Mechanical tactile stimulation was provided to the right index finger pad

**Fig. 5:**
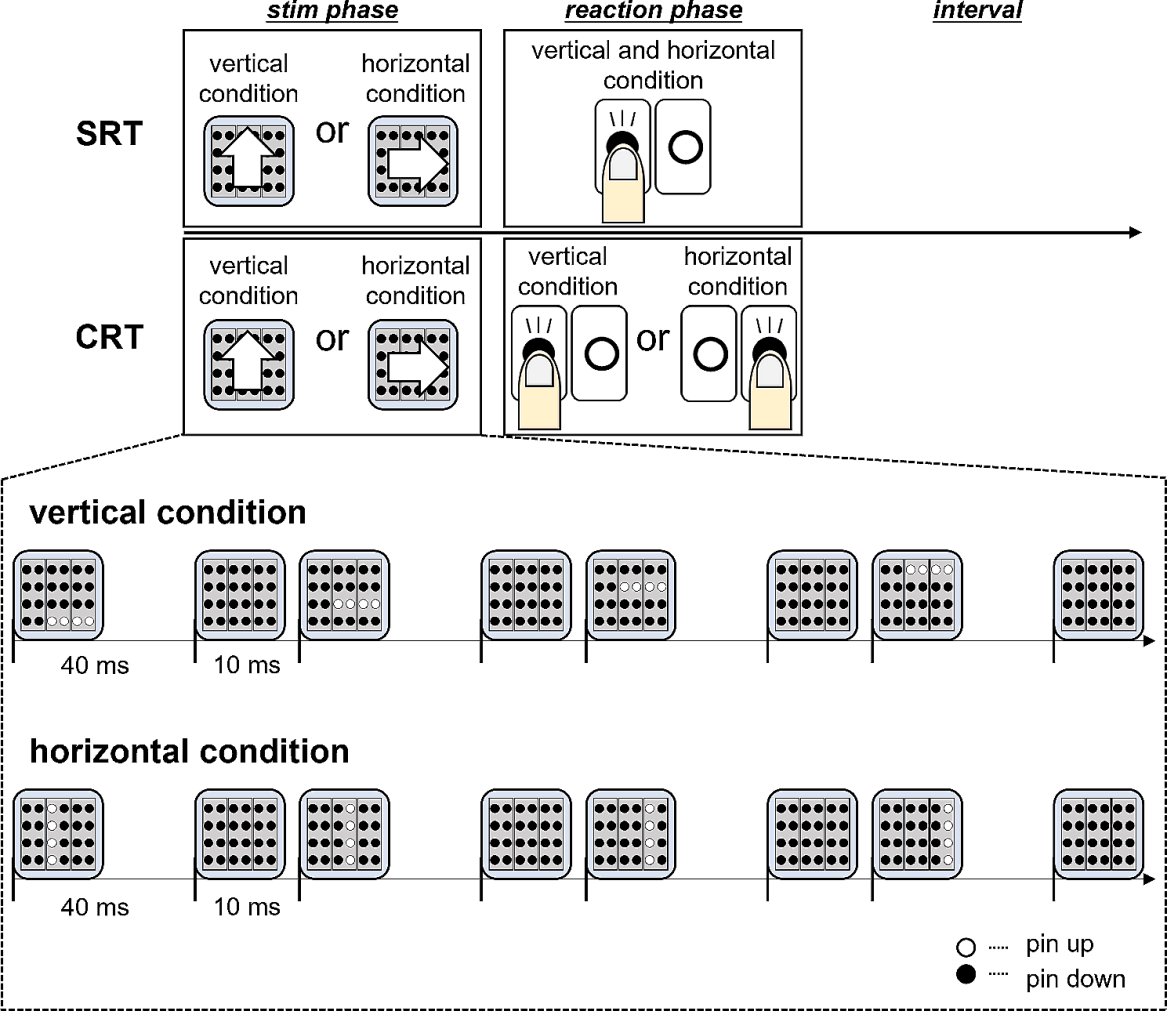
Paradigm of simple reaction task and choice reaction task. The time for a row of stimulation pins to appear was 40 ms, the time for all pins to disappear was 10 ms, and the time required for the presentation of one tactile stimulation trial was 200 ms. The stimulation patterns in the conditions wherein the four pins in a horizontal row moved vertically from proximal to distal on the right index finger and wherein four pins in a vertical row moved horizontally from left to right across the right index finger

At the beginning of the task, the participants sat in a chair, relaxed, and wore earplugs. Tactile discrimination is reportedly improved gazing at the stimulation site [14, 15]. Therefore, the participants looked at the fixation point’s displayed center of the monitor placed in front during the task. The distance between the eyebrows and fixation point was 40 cm. MS was delivered by a piezoelectric actuator (TI-1101; KGS, Saitama, Japan) moving 16 plastic pins. These plastic pins are 1.3 mm in diameter, with a projection height of 0.8 mm and a pushing force of 0.031–0.12 N/pin. The tactile stimulation’s target area was the right index finger pad (Fig. 4). There were two stimulation pattern conditions (Fig. 5). In one condition, four pins in a horizontal row were moved in the vertical (proximal to distal) direction within the stimulation area (vertical condition). In the other condition, four pins in a vertical row were moved in the horizontal (left to right) direction within the stimulation area (horizontal condition). The “up” phase of the periodic stimulus, when the stimulation pins were protruding, was 40 ms, whereas the “down” phase, when all 16 pins were withdrawn, was 10 ms. The trial time measured from the moment the first row of pins appeared until the last row of pins was withdrawn was 200 ms. Therefore, the “down” phase was set to 10 ms to make the participants perceive each row of stimulation pins as a continuous stimulation. The intertrial presentation interval was 4000–5000 ms. In the SRT and CRT, vertical and horizontal trials order were randomly presented.

In the SRT, a button should be pressed as quickly as possible with the left middle finger in response to a stimulation presented to the right index finger pad. The participants were instructed to press the button as quickly as possible in response not to the direction of the stimulation movement but to its presentation. The SRT comprised 25 times of vertical condition stimulation and 25 times of horizontal condition stimulation, totaling to 50 times of stimulation. The time taken from stimulation presentation to button response was recorded as RT.

The CRT is the discrimination vertical from the horizontal conditions of the MS moving direction. The participants pressed the button with their left middle and left index fingers for MS with a vertical and horizontal condition stimulations, respectively. The participants were instructed to discriminate and react as quickly and accurately as possible. The CRT comprised 50 times of vertical condition stimulation and 50 times of horizontal condition stimulation, totaling to 100 times of stimulation. The RT and correct rate were recorded.

### MS intervention

The intervention conditions were as follows: RGS, all 16 pins in the stimulation plane simultaneously and repeatedly up and down (Fig. 1A); HMS, four plastic pins in a vertical row regularly and repeatedly appear and disappear from left to right (Fig. 1B); and Control, no stimulation (Fig. 1C). The MS intervention was applied for 20 min. The on/off cycle of the MS intervention comprised 1 s of stimulation on and 5 s of stimulation off. During the period of the stimulation, MS was presented at a frequency of 20 Hz. comprising 10-ms pins up and 40-ms pins down. This study used a crossover design of three repetitions of the experiment; thus, participants performed one intervention condition per experiment. The order of each intervention condition was randomized across participants. The interval between each condition was at least 5 days.

### Data and statistical analyses

According to previous studies, all trials with RT < 50 ms were excluded to eliminate reactions due to prediction in the SRT analysis [27], and the mean RTs was calculated and compared before and after each intervention. For the CRT analysis, only the trials with the RT values between 50 and 1500 ms were included, whereas the trials with RT < 50 or > 1500 ms were excluded from the analysis. The mean RTs, correct rate, RCS [17] were calculated for each intervention condition and the data obtained before and after the intervention were compared for each intervention condition. The RCS is the number of correct responses divided by the sum of RT of all trials, including wrong responses. The RCS is a sensitive measure of the speed-accuracy tradeoff [18] and has been adopted in studies recording RT and correct rate [19, 20]. Statistical analysis was performed using SPSS statistics 24 software (IBM SPSS, Armonk, NY, USA). The RT of the SRT and RT, correct rate, and RCS of the CRT were compared before and after the intervention. The Shapiro–Wilk test was used to test normality. Therefore, we used GLMM to analyze the main effect of the intervention condition (RGS, HMS, and Control interventions) and time (pre, post), interaction, and random effects of the participants.

Given that the RCS in the CRT did not show normality, the Wilcoxon signed-rank test was conducted. We used “r” as the effect size. Statistical significance was set at a P-value of < 0.05.

## Data Availability

The datasets generated for this study are available on request to the corresponding author.

## Abbreviations

S1: Primary somatosensory cortex
S2: Secondary somatosensory cortex
MS: Mechanical tactile stimulation
LTP: Long-term potentiation
IPC: Inferior parietal cortex
SRT: Simple reaction task
CRT: Choice reaction task
RT: Reaction time
RCS: Rate correct score
RGS: Repetitive global tactile stimulation
HMS: Horizontal moving tactile stimulation
GLMM: Generalized linear mixed model
